# RhoTermPredict: an algorithm for predicting Rho-dependent transcription terminators based on *Escherichia coli*, *Bacillus subtilis* and *Salmonella enterica* databases

**DOI:** 10.1186/s12859-019-2704-x

**Published:** 2019-03-07

**Authors:** Marco Di Salvo, Simone Puccio, Clelia Peano, Stephan Lacour, Pietro Alifano

**Affiliations:** 1 Department of Biological and Environmental Sciences and Technologies, University of Salento, Lecce, Italy; 2 Humanitas Clinical and Research Center, Rozzano, Milan Italy; 3 Institute of Genetics and Biomedical Research UoS of Milan, National Research Council, Rozzano, Milan Italy; 4 Univ. Grenoble Alpes, CNRS, Inria, LIPhy (UMR5588), 38000 Grenoble, France

**Keywords:** RhoTermPredict, Rho-dependent terminators, RUT site, Motif, Transcription termination, Rho

## Abstract

**Background:**

In bacterial genomes, there are two mechanisms to terminate the DNA transcription: the “intrinsic” or Rho-independent termination and the Rho-dependent termination. Intrinsic terminators are characterized by a RNA hairpin followed by a run of 6–8 U residues relatively easy to identify using one of the numerous available prediction programs. In contrast, Rho-dependent termination is mediated by the Rho protein factor that, firstly, binds to ribosome-free mRNA in a site characterized by a C > G content and then reaches the RNA polymerase to induce its release. Conversely on intrinsic terminators, the computational prediction of Rho-dependent terminators in prokaryotes is a very difficult problem because the sequence features required for the function of Rho are complex and poorly defined. This is the reason why it still does not exist an exhaustive Rho-dependent terminators prediction program.

**Results:**

In this study we introduce RhoTermPredict, the first published algorithm for an exhaustive Rho-dependent terminators prediction in bacterial genomes. RhoTermPredict identifies these elements based on a previously proposed consensus motif common to all Rho-dependent transcription terminators. It essentially searches for a 78 nt long RUT site characterized by a C > G content and with regularly spaced C residues, followed by a putative pause site for the RNA polymerase. We tested RhoTermPredict performances by using available genomic and transcriptomic data of the microorganism *Escherichia coli* K-12, both in limited-length sequences and in the whole-genome, and available genomic sequences from *Bacillus subtilis* 168 and *Salmonella enterica* LT2 genomes. We also estimated the overlap between the predictions of RhoTermPredict and those obtained by the predictor of intrinsic terminators ARNold webtool. Our results demonstrated that RhoTermPredict is a very performing algorithm both for limited-length sequences (F_1_-score obtained about 0.7) and for a genome-wide analysis. Furthermore the degree of overlap with ARNold predictions was very low.

**Conclusions:**

Our analysis shows that RhoTermPredict is a powerful tool for Rho-dependent terminators search in the three analyzed genomes and could fill this gap in computational genomics. We conclude that RhoTermPredict could be used in combination with an intrinsic terminators predictor in order to predict all the transcription terminators in bacterial genomes.

**Electronic supplementary material:**

The online version of this article (10.1186/s12859-019-2704-x) contains supplementary material, which is available to authorized users.

## Background

In addition to initiation, transcription termination represents the other essential “punctuation marks” for DNA transcription and, hence, an important regulatory step of gene expression. In bacteria, the DNA transcription can terminate through two different mechanisms: the Rho-independent or “intrinsic” termination and the Rho-dependent termination [1]. Intrinsic terminators are characterized by an RNA structure having a GC-rich hairpin immediately followed by a stretch of 6–8 uridine residues [1, 2], while Rho-dependent terminators rely upon the interaction of a protein called Rho with the RNA Polymerase (RNAP) [1, 3–5]. Bacterial Rho is a hexameric RNA-DNA helicase that serves as a general bacterial transcription termination factor [5, 6]. Rho binds preferentially to unstructured and ribosome-free C-rich and G-poor nascent RNA, of at least 70–80 nt, with regularly spaced cytosines [1, 3, 5, 7]. This site is known as the Rho utilization site (the so-called RUT site). The depletion of G within a natural RUT site minimizes the formation of potentially interfering secondary structures, which generally inhibit Rho binding [1, 8–10].

After the binding with the RUT site, Rho traverses RNA in the 5′ to 3′ direction via RNA-dependent ATP hydrolysis, all the while threading RNA through its central cavity [3, 5, 6, 8–10]. According to a widely accepted model, Rho catches up to the elongation complex by translocating along the nascent transcript and, at certain pausing sites, dissolves the elongation complex by pulling out the transcript [1, 3, 4, 6, 11]. Allosteric interactions between Rho and RNAP facilitating catalytic inactivation and eventual dissociation of the elongation complex have been also more recently proposed [11, 12]. The site of termination is typically within a window of 10–20 nt downstream of the RUT site [1, 13], and it is rarely more than 100 nt downstream.

Over the past decade, a lot of studies, performed in several bacterial species, have established the importance of Rho in gene regulation and its conserved role in the enforcement of transcription-translation coupling, by interrupting transcription of untranslated mRNAs [14–16]. Furthermore, in *Escherichia coli*, *Bacillus subtilis*, *Staphylococcus aureus*, and *Mycobacterium tuberculosis* an important role of Rho in suppression of pervasive, primarily antisense transcription was demonstrated [17–20]. Complete or even partial inactivation of Rho in these bacterial species causes widespread transcription originating from cryptic promoters and read-through of transcription terminators [21].

Intrinsic terminators can be identified using bioinformatics approaches with on line algorithms/tools. Among them, currently available and most performing tools for prokaryote intrinsic terminator prediction include TransTermHP [22], RNIE [23], the commercial program Softberry’s FindTerm [24] and ARNold [25]. Conversely, Rho-dependent terminators have so far proved difficult to predict computationally because the sequence features required for the function of Rho are complex and poorly defined, in contrast to intrinsic terminators features [1]. In fact, only recently the first prediction model for Rho-dependent termination of transcription was proposed [26], but it was designed specifically for predicting only RUT sites, omitting the presence of RNAP pausing sites where Rho induces RNAP release from RNA. Hence at the moment, other Rho-dependent terminators prediction programs that take into account all the steps of the Rho-dependent transcription termination have not yet been created.

Rho is very often present in bacterial genomes and the basic principles of Rho-dependent-termination are conserved across species, despite some structural differences between Rho proteins [21]. About 20–30% of the transcription terminators identified in bacterial genomes are Rho-dependent, even about half in *E. coli* [3]. For this reason the implementation of an algorithm for the prediction of terminators mediated by Rho factor could be certainly very useful.

In this study we introduce RhoTermPredict, a novel algorithm for the prediction of transcriptional Rho-dependent terminators in *E. coli*, *B. subtilis*, *Salmonella enterica* and eventually other bacterial genomes. RhoTermPredict is the first program implemented for an exhaustive search of Rho-dependent terminators, which functions in two steps to specifically identify this type of transcription termination sites within a genome sequence. Our aim was to create a program for the prediction of such elements in a prokaryotic genome based on a conserved structured motifs search, in a similar way to our previous work regarding the promoter prediction algorithm G4PromFinder [27]. This novel algorithm searches for proposed C > G content RNA motif [1, 5, 9, 28] as possible C-rich element in Rho-dependent transcription terminators, followed by a possible pause site for RNAP. We tested RhoTermPredict performances by using available genomic and transcriptomic data of the model microorganism *E. coli* K-12 and a list of Rho-dependent terminators obtained by [18]. In order to estimate the degree of overlap between Rho-dependent and intrinsic terminators predictions, we also run one of the currently available tools for bacterial intrinsic terminators prediction on the same sets of *E. coli* K-12 genome used for the development of RhoTermPredict. We decided to use ARNold tool [29] because it is, at the moment, the only freely accessible online tool very simple to use, available for finding intrinsic terminators in a raw DNA/ RNA sequence [25]. ARNold searches for intrinsic terminators using two complementary programs, Erpin [30] and RNAmotif [31]. ARNold takes as input DNA or RNA sequences in fasta format and provides as output the 5′ end position of predicted transcription terminator, the strand, the terminator sequence and the free energy of stem-loop region. In addition to *E. coli* K-12, we tested RhoTermPredict on some available genome sequences from *B. subtilis* 168 [17] and *S. enterica* LT2 [26].

The RhoTermPredict algorithm described in this study is available from: https://github.com/MarcoDiSalvo90/RhoTermPredict.

## Implementation

### Programming language and data sets

RhoTermPredict algorithm was written in the Python (v.3.6) language [32], and requires libraries BioPython, numpy, re and openpyxl. It accepts as input bacterial genome sequences, and provides as output the coordinates of putative Rho-dependent terminators elements (RUT and RNAP pause sites) with a score assigned to them that indicates the probability that the extracted region actually corresponds to a Rho-dependent terminator (see below for the scoring assignment method). For Rho-dependent terminator predictions, we used available genomic sequences of the model microorganism *E. coli* K-12 substr. MG1655 (National Center for Biotechnology Information, accession code NC_000913.3) (see below). For the prediction quality evaluation, we used *E. coli* K-12 genomic annotation [33] together with relative RNA-Seq data (see below) and a list of Rho-dependent transcription terminators obtained by [18] (see below). In addition to *E. coli* K-12, with the purpose of evaluating the algorithm performances on other bacterial genomes, we run RhoTermPredict on genome sequences from *B. subtilis* 168 (NC_000964.3) [17]. In fact, although the transcription termination mechanisms in *B. subtilis* have remained poorly defined for a long time, recently the action of Rho has been verified in it [17, 21]. The procedure to search for putative Rho-dependent terminators is reported below.

### Procedure to search for putative rho-dependent terminators

RhoTermPredict actually searches for some mandatory elements (putative RUT and RNAP pause sites) and for other optional elements whose presence could increase the prediction score. RhoTermPredict search is not based on “nucleotide sequence homology”, but rather on “conserved motifs in the nucleotide sequences”, and these motifs were inferred from literature data on RUT sites and on RNA Polymerase pausing sites. In fact, a consensus motif common to all Rho-dependent transcription terminators has been previously proposed in *E. coli* and *S. enterica* [5, 9, 28].

**Fig. 1:**
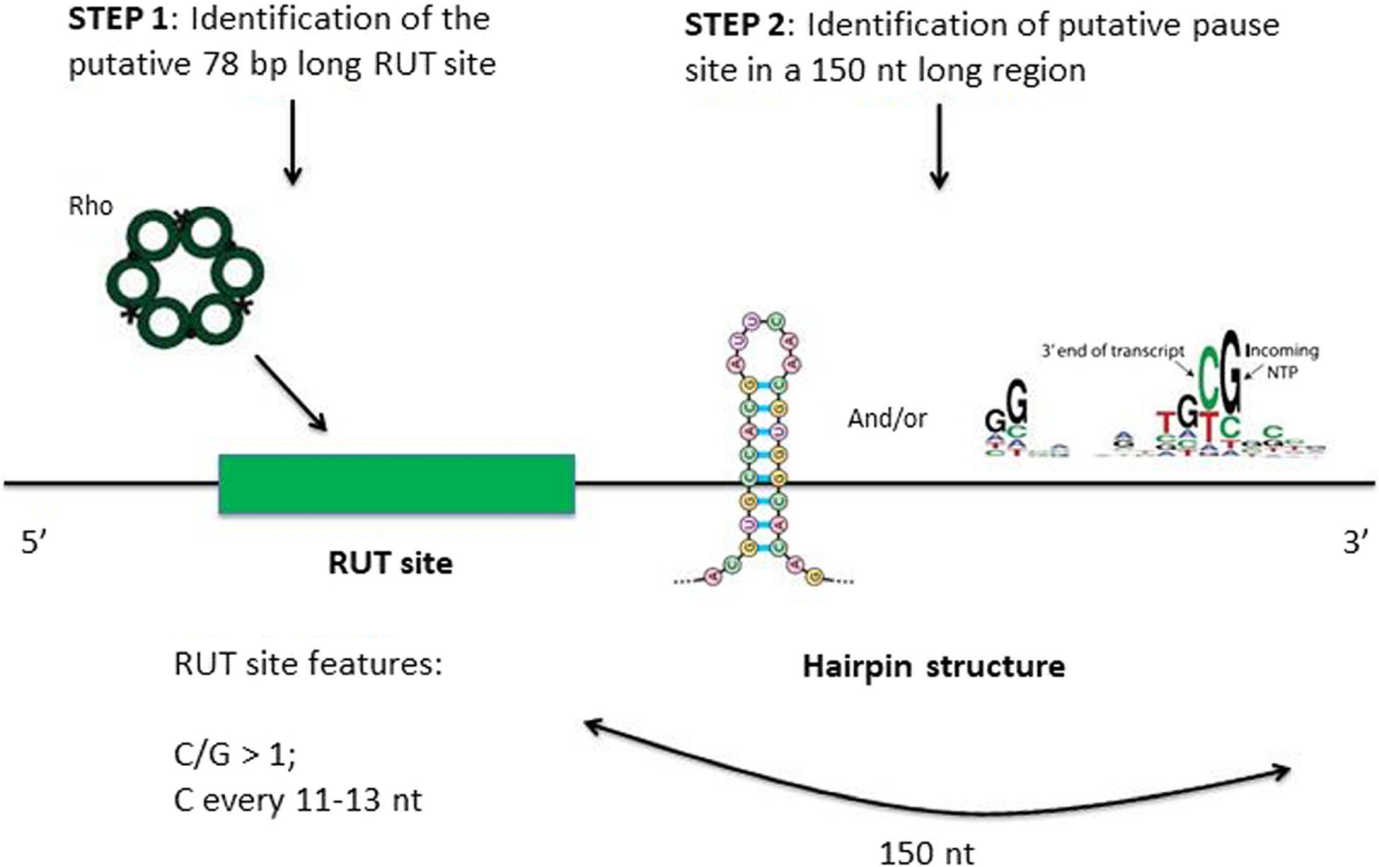
Method used for the prediction of putative Rho-dependent terminators. The adopted procedure is constituted by 2 steps: i) the identification of the RUT site and ii) the identification of the RNAP pausing site in a 150 nt long region immediately downstream from the predicted RUT site 3′

On that basis, we elaborated the following two-step procedure to detect Rho-dependent terminators (Fig. 1): i.) The first step is the identification of the putative “RUT site”. To do this, the algorithm scans a window of 78 nt over the query sequence, one nt at a time, until the C/G ratio of the window exceeds the threshold value of 1 and with regularly spaced cytosine residues within the window (every 11–13 nt). Then, by scanning a window of 128 nt (starting from the previous position where the C/G content of the window reached a value > 1), the 78 nt long region with maximal C/G content and with regularly spaced cytosine residues (herein referred to as RUT site) is selected. Therefore RhoTermPredict searches for a putative 78 nt long RUT site characterized by a maximal C/G content (in any case greater than 1) localized in a 128 nt long region (128 stands for 78 + 50: 78 is the extension of the RUT site, 50 an arbitrary value chosen to maximize the C/G content of the RUT site). This consensus motif and its extension (78 nt) fit well with structural and functional properties of Rho hexamer and its interaction with C-rich RNA sequences at the level of its primary RNA binding domain [5, 34–36]. This binding leads to the positioning of the RNA into the secondary RNA binding domain, which in turn activates the ATPase for its translocase/RNA–DNA helicase functions [6, 37]. ii.) The second step is the identification of a putative pause site for RNAP in a region extended up to 150 nt downstream from the 3′-end of the selected RUT site. The RNAP pause sites searched were hairpin structures [38, 39] (with a GC-rich stem and a loop constituted by 4–8 nt). Alternatively to hairpin structures, in the same 150 nt long region, we considered as putative RNAP pause site the presence of the consensus pause-inducing sequence element G_− 11_G_− 10_(C/T)_− 1_G_+ 1_ (where −1 corresponds to the position of the RNA 3′ end) identified by [40, 41]. However, the presence of the previous element close to a putative hairpin structure (precisely within the hairpin structure extended up to 5 nt upstream from its 5′ end and 5 nt downstream from its 3′ end) provides a higher score to the prediction.

RhoTermPredict also allows to predict multiple putative terminators in a single query region, and to search for terminators in both strands.

### Procedure for rho-dependent terminator scoring assignment

The maximum score that could be assigned by our algorithm to a terminator prediction is 15, while the minimum is 6 (a minimum of 3 point for the RUT site and also a minimum of 3 points for a pause site). An addition of 1 point is assigned if the C/G ratio of RUT site > 1.25, of 2 points if such ratio > 1.5, of 3 if > 2. Regarding to the hairpin structure as predicted pause site, an other point is attributed if the GC-content of the hairpin stem is > whole genome GC-content + 10, instead 2 points if it is > whole genome GC-content + 20 (because it is known that the hairpin stem is GC-rich) while an extra 0.5 point it is assigned if the hairpin loop length is < 6 nt or if the hairpin stem length > 4. Finally 3 extra points are assigned if the consensus pause-inducing sequence element G_−11_G_− 10_(C/T)_−1_G_+ 1_ is present near the putative hairpin pause site.

### Dataset of rho-dependent terminators, and construction of positive and negative sequence datasets

To test the reliability of RhoTermPredict predictions we used a total of 1264 regions containing Rho-dependent termination sites (defined as “BCM significant transcripts”, BSTs) obtained by [18] growing up the microorganism *E. coli* K-12 with or without the specific Rho inhibitor bicyclomycin at a concentration that reduces Rho function without affecting the rate of cell growth. Therefore a differential expression of these BSTs regions indicates the presence of a Rho-dependent transcription terminator.

Starting from the genome of *E. coli* K-12 (NC_000913.3) and the just-indicated BSTs regions, we generated a Rho-dependent terminator set (positive set) consisting of 300 nt long sequences immediately upstream all the BSTs regions. The positive set consisted of 1264 sequences. Instead, we constructed the negative set with all the intergenic regions (IRs) < = 300 nt and > = 200 nt in length in which terminators were not expected. To do this, we considered all the IRs that separated two divergently oriented coding sequences (CDSs). The negative set consisted of 195 sequences.

For *B. subtilis* 168 we produced a positive and a negative set of sequences in the same way as for *E. coli* K-12. In this case, the positive set was represented by 34, 300 nt-long, sequences that were immediately upstream from the genomic regions, obtained by [17], relative to extended mRNAs in the mutant strain of *B. subtilis* 168 lacking the termination factor Rho. As for *E. coli* K-12, as a negative set of sequences we considered all the IRs < = 300 nt and > = 200 nt in length separating two divergently oriented CDSs. For *B. subtilis* 168, the negative set consisted of 149 sequences.

### Estimation of RhoTermPredict performances

RhoTermPredict performances were evaluated by using the following statistical measures:

$$ \mathrm{Recall}\ \left(\mathrm{sensitivity}\ \mathrm{or}\ \mathrm{the}\ \mathrm{true}\ \mathrm{positive}\ \mathrm{rate}\right)=\mathrm{TP}/\left(\mathrm{TP}+\mathrm{FN}\right) $$

$$ \mathrm{Precision}\ \left(\mathrm{the}\ \mathrm{positive}\ \mathrm{predictive}\ \mathrm{value}\right)=\mathrm{TP}/\left(\mathrm{TP}+\mathrm{FP}\right) $$

$$ \mathrm{Specificity}\ \left(\mathrm{the}\ \mathrm{true}\ \mathrm{negative}\ \mathrm{rate}\right)=\mathrm{TN}/\left(\mathrm{TN}+\mathrm{FP}\right) $$

$$ \mathrm{Accuracy}\ \left(\mathrm{the}\ \mathrm{fraction}\ \mathrm{of}\ \mathrm{samples}\ \mathrm{correctly}\ \mathrm{classified}\right)=\left(\mathrm{TP}+\mathrm{TN}\right)/\left(\mathrm{TP}+\mathrm{TN}+\mathrm{FP}+\mathrm{FN}\right) $$

$$ {\mathrm{F}}_1-\mathrm{score}\ \left(\mathrm{the}\ \mathrm{harmonic}\ \mathrm{mean}\ \mathrm{of}\ \mathrm{Precision}\ \mathrm{and}\ \mathrm{Recall}\right)={2}^{\ast }{\mathrm{Precision}}^{\ast}\mathrm{Recall}/\left(\mathrm{Precision}+\mathrm{Recall}\right) $$

where TP = True positives, FP = False positives, FN = False negatives and TN = True negatives.

We considered as either true positive (TP) or false positive (FP) any sequences of either the positive or the negative set in which the algorithm predicted a terminator, respectively. Importantly, at most one TP was considered for each sequence of the positive set. We considered as either true negative (TN) or false negative (FN) any sequences of either the negative or the positive set in which the algorithm did not predict any terminator, respectively.

### RNA-sequencing, reads mapping quality assessment

Ribosomal RNAs were depleted using RiboZero Gram negative kit (Epicentre,Illumina) and strand-specific sequencing libraries were constructed using the ScriptSeqTM v2 RNAseq library preparation kit. After that, the purified cDNA library was sequenced on an Illumina GAIIx/Solexa or MiSeq platform (Illumina, San Diego, CA) with 76-bp paired-end reads. The BAM files for each condition analysed are publicly available at Sequence Reads Archive (SRA) under accession number BioProject PRJNA483864. Alignment to the reference strain of *E. coli* K-12 genome (Ref Seq NC_000913) was done using bowtie2 (with sensitive options, corresponding to -D 15 -R 2 -L 22 -i S,1,1.15; see the Bowtie2 manual for the explanation of the flags –D,-R,-L,S, http://bowtie-bio.sourceforge.net/bowtie2/manual.shtml#reporting). Evaluation of strand specificity and gene coverage was performed using BEDTools (v2.20.1) and SAMtools (v0.1.19). Wig files were generated from the aligned BAM files by using BEDTools (v2.20.1). To avoid bias caused by multi-mapping reads the non-deterministic option and end-to-end mode were used to force a single assignment of multi-mapping reads to the best scoring region (if present) or in the case of regions with identical scores reads were randomly assigned. Mapped reads with MAPQ (mapping quality) greater than 30 were analyzed to determine the read counts per protein-coding gene.

### Genome-wide analysis and validation with RNA-Seq data

We also run RhoTermPredict on the whole genome of *E. coli* K-12 to test its genome-wide predictive performances. To validate these predictions, we used the RNA-Seq data above described. We considered the predicted regions as putative Rho-dependent terminators if they were in positions in which there was a negative transcription gradient in RNA-Seq data, as suggested in other works [17, 21].

Precisely, we considered, in this case, a prediction as TP if there was a decrease of read value by a factor of at least 1.5 between the read value at the putative RUT site 5′-end point and the read count value 150 nt downstream from putative RUT site 3′-end point (hence after the putative RNAP pause site), the first with a read count value ≥10. We considered this constraint in order to analyze only the predictions near an expressed DNA region in the RNA-Seq data. In fact, a prediction close to a not-expressed DNA region could be incorrectly considered as a FP.

## Results

In this study we used as data sources: i.) the available genome sequences of *E. coli* K-12 [33] for Rho-dependent terminator prediction; ii) the BSTs regions obtained by [18] (see Implementation section) and RNA-Seq data (this study) for terminator prediction validation. The complete genome of *E. coli* K-12 has a GC-content of 50.79%, and consists of putative 4518 genes. The results obtained are shown below. Moreover, we decided to test RhoTermPredict also on genome sequences of *B. subtilis* 168 in order to analyze RhoTermPredict performances on another genome different from *E. coli* K-12.

### Rho-dependent terminators prediction by RhoTermPredict in *E. coli* K-12 and evaluation

**Fig. 2:**
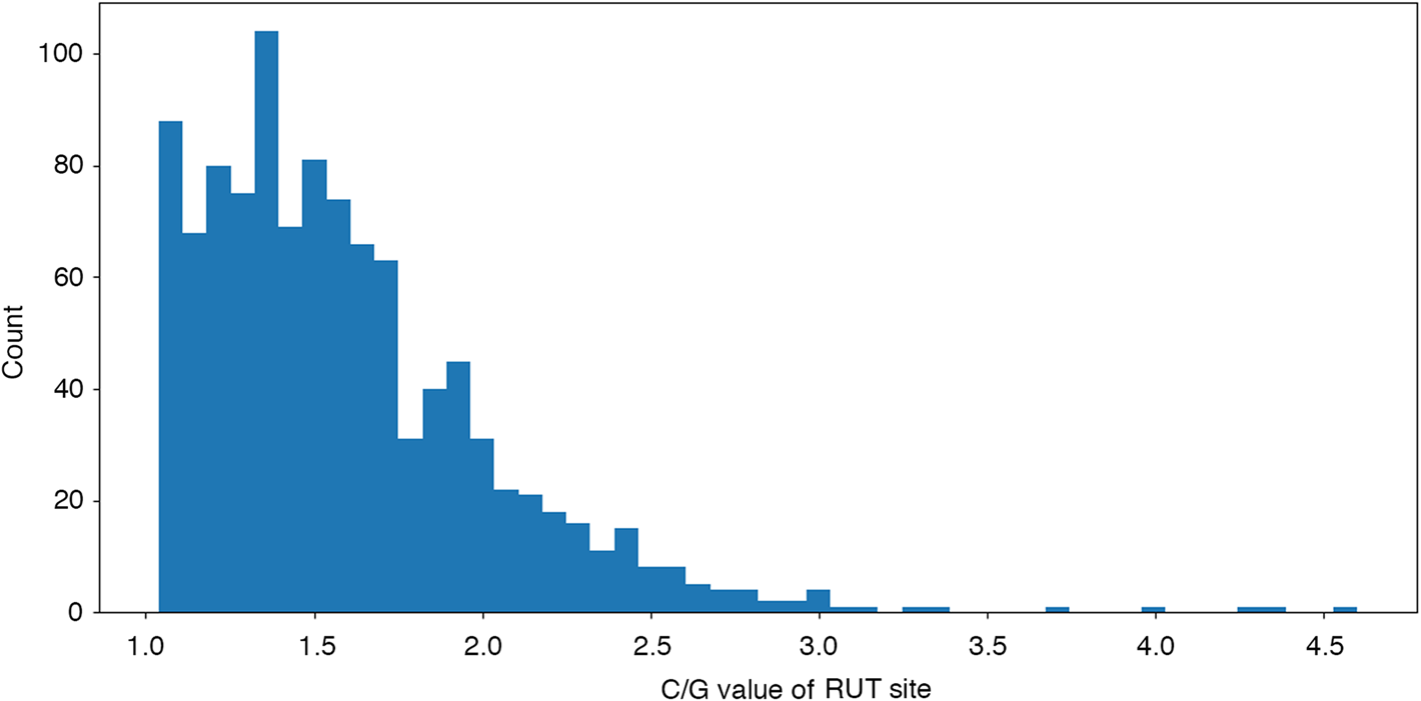
Distribution of C/G content of predicted terminators RUT sites

**Fig. 3:**
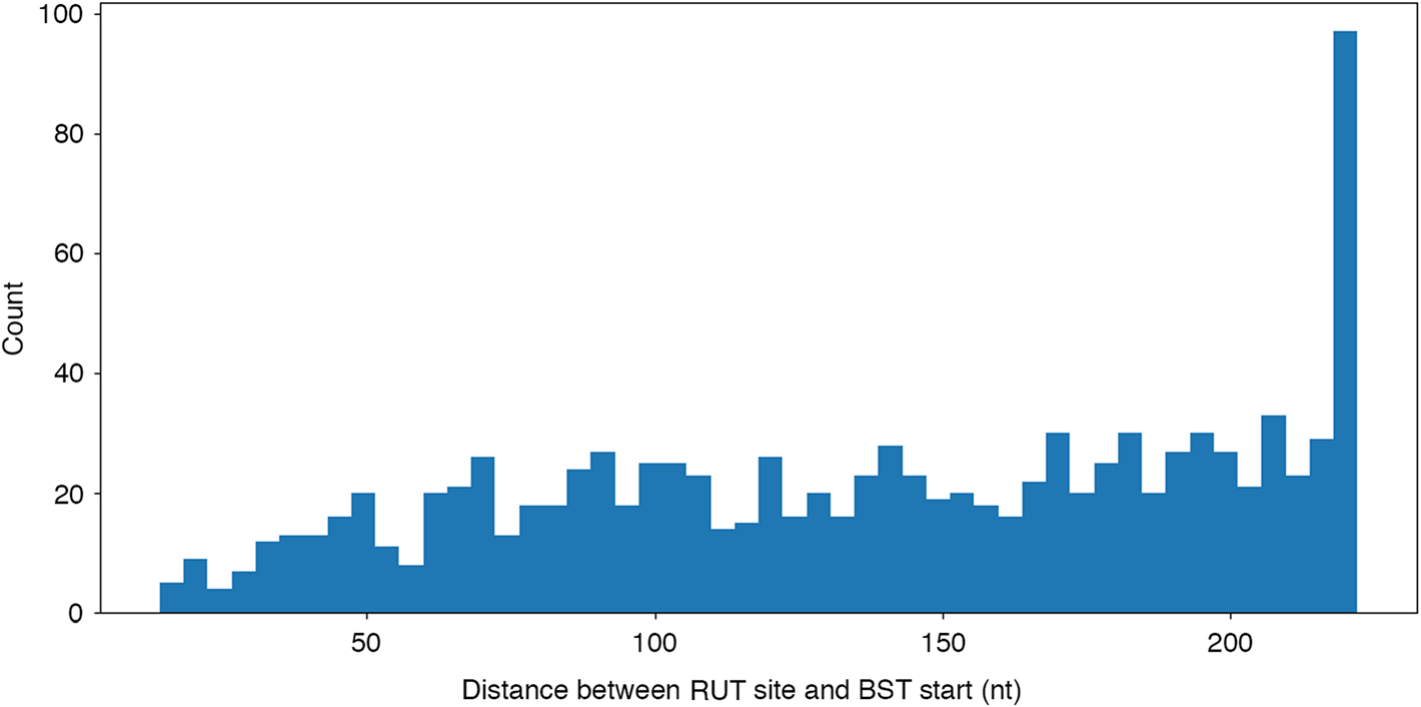
Distribution of predicted Rho-dependent terminators RUT sites in *E. coli* K-12 as a function of their distance from the BST regions. Predicted Rho-dependent terminators are grouped based on distances between the RUT site 3′-end points and the annotated BST regions 5′-end points

Table 1 summarizes statistics of putative Rho-dependent terminators that were predicted by RhoTermPredict in the positive set. All predicted terminators without any limitation on the prediction score were taken into account. In the positive set of sequences, the algorithm predicted putative terminators in most (64.5%) of the analyzed regions (Table 1). A total of 1064 putative terminators were predicted. Multiple putative terminators were sometime associated with single sequences of the positive set. In particular, in 17.2% of examined regions, more than one predicted terminator could be found within the 300 nt of the positive set of sequences. In Fig. 2 we show the distributions of C/G content of predicted RUT sites terminators (the mean value is 1.6, as reported in Table 1), while in Fig. 3 we show the distributions of distances occurring between the 3′-end points of Rho-dependent terminators RUT sites and the 5′ end points of relative annotated BST regions.

**Table 1:** Statistics of predicted Rho-dependent terminators in the *E.coli* K-12 genome sequences of the positive set by RhoTermPredict algorithm

Positive dataset size	Regions with at least one prediction (%)	Regions with more predictions (%)	Total number of predictions	Mean C/G content of RUT site
1264	64.5	17.2	1064	1.6

Then we evaluated RhoTermPredict performances on terminator prediction using a positive set of sequences including the 300 nt long regions immediately upstream of all the BSTs regions, and a negative set of sequences represented by IRs of *E. coli* K-12 genome, located between two divergently oriented CDSs with length between 200 and 300 nt (see Implementation section for details). To use without bias the positive and negative sequence datasets, which originally did not have the same size, we randomly selected 195 sequences (negative set size) of the positive sets, and performed the tests 10 times with different series of randomly selected sequences to obtain mean values (Table 2, columns 1 and 2). In this way, we used a positive and a negative set having the same size. We also reported in Table 2 the results, obtained in the same positive and negative sets, by ARNold Rho-independent terminators prediction tool [25, 29]. We saw RhoTermPredict well performing with the analyzed genome. The F_1_-score with our algorithm was about 0.7, recall was 65.6%, precision was 73.6% and, finally, specificity and accuracy were, respectively, 76.4 and 71.0% (Table 2). As expected, ARNold, being an intrinsic terminators prediction tool, was not performing in the prediction of terminators in genomic regions where Rho-dependent terminators were expected (F_1_-score very low, about 0.1, Table 2). Therefore the degree of overlap between RhoTermPredict and ARNold predictions (and, hence, between Rho-dependent and intrinsic terminators) in the previous regions was very low.

**Table 2:** Testing results of RhoTermPredict and performances of the Rho-independent terminators tool ARNold^a^ in the positive and negative set of sequences

Tool	TP	FN	FP	TN	Precision (%)	Recall (%)	Specificity (%)	Accuracy (%)	F_1_-score
RhoTermPredict	128	67	46	149	73.6	65.6	76.4	71.0	0.7
ARNold	11	184	19	176	36.7	5.6	90.3	48.0	0.1

^a^Test experiments were repeated 10 times for 195 randomly selected sequences of positive sets of *E. coli* K-12, and the means were taken

### Genome-wide analysis in *E. coli* K-12

**Fig. 4:**
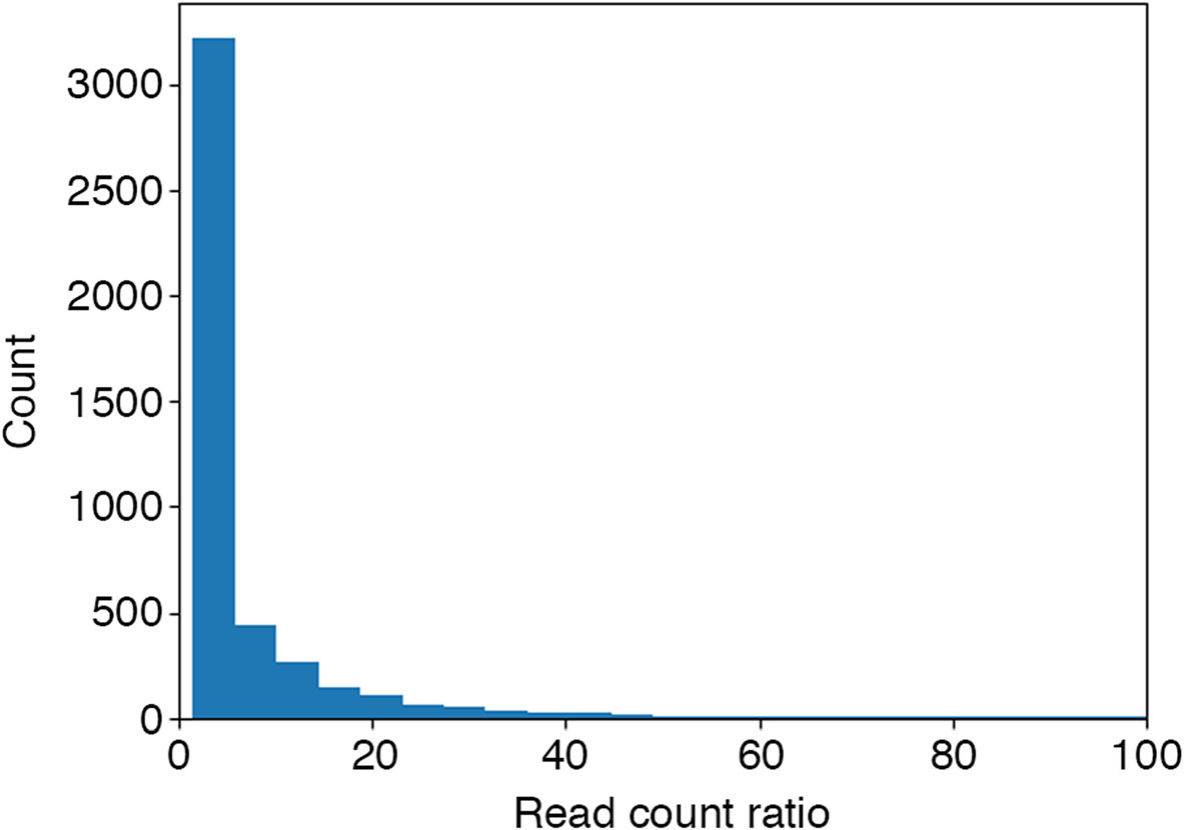
Distribution of RNA-Seq read values ratios between the read value of putative RUT site 5′-end point and the read value 150 nt downstream from putative RUT site 3′-end point of validated genome-wide predictions

We evaluated RhoTermPredict performances not only with sequences of limited length, but also with the whole genome sequence of *E. coli* K-12. Definitely, we performed a genome-wide analysis, in order to assess if RhoTermPredict is only performing with sequences of limited length or even with whole genomes. Overall, RhoTermPredict predicted a total of 23,930 (839 in IRs) putative Rho-dependent terminators (Additional files 1 and 2) in the *E. coli* K-12 genome (Table 3). Of these, only 7200 (319 in IRs) were next to transcribed DNA regions that we used for the genome-wide prediction validation (see Implementation section). Precisely, Rho-dependent terminator predictions were validated by RNA-Seq data at a percentage of 62.4% (70.5% in IRs). In Fig. 4 we show the distribution of the read values ratios between the two ends (see Implementation section for details) of validated predictions (the mean value was about 11.8). We could see the read value ratios as a measure of the terminator strength.

**Table 3:** Statistics of predicted Rho-dependent terminators in the *E. coli* K-12 whole-genome and IRs by RhoTermPredict algorithm and evaluation with RNA-Seq data

Dataset	Total number of predictions	Predictions next to expressed DNA regions	Validated predictions (%)
Whole genome	23,930	7200	62.4
IRs	839	319	70.5

As there is evidence that termination efficiency at the level of Rho-dependent terminators may depend on a number of factors such as intrinsic characteristics of the RUT site, C/G ratio and kinetic coupling between RNA polymerase and Rho [9, 28, 42–44], we performed correlation analysis between the previous RNA-Seq read value ratios and C/G ratios at the level of the putative RUT site of Rho-dependent terminators that were identified by the algorithm (Additional file 3: Figure S1). The analysis revealed no strong correlation between C/G ratio and RNA-Seq read value ratios, but, interestingly, all the read value ratios > 100 (precisely 46) were obtained for putative RUT sites with a C/G content < 2.The mean value of the read value ratios for predictions with a RUT site C/G content < 2 was about 12.1, 7.9 for predictions with a RUT site C/G content > = 2. This finding was quite unexpected and could bring new mechanistic information.

### Evaluation of overlap’s degree between RhoTermPredict and ARNold genome-wide predictions

In order to have a further demonstration that RhoTermPredict algorithm is specific on searching for Rho-dependent terminators, we evaluated the overlap’s degree between RhoTermPredict and ARNold genome-wide predictions with the *E. coli* K-12 genome. For this analysis, we considered all the 23,930 genome-wide predictions of RhoTermPredict (Additional file 1). ARNold tool, instead, predicted a total of 3190 putative intrinsic terminators in the same whole genome. We considered a RhoTermPredict prediction overlapping with an ARNold prediction if they were within 50 nt from each other. 50 nt is an arbitrary value that we used because two predicted terminators that are within 50 nt from each other may actually represent the same element especially if we consider that the transcription termination is often heterogeneous [26]. Overall, we obtained a total of 751 overlapping predictions of the two programs, i.e. about 23% of ARNold predictions overlapped with a RhoTermPredict prediction. However, some degree of overlapping is expected due to the frequent occurrence of hairpin structure(s) downstream of RUT sites.

### Rho-dependent terminators prediction by RhoTermPredict in *B. subtilis* 168 and evaluation

The results obtained by RhoTermPredict and ARNold for the genomic regions of the positive and the negative set of *B. subtilis* 168 were reported in Table 4. As for *E. coli* K-12, we decided to use a positive and a negative set with the same size, to fairly compare them. In fact the original size of the positive and the negative set was, respectively, 34 and 149 (see Implementation). With this aim, we randomly selected 34 regions (positive set size) of the negative set, and we repeated the testing 10 times on different series of randomly selected sequences to obtain the mean values reported in Table 4. In this way, we used a positive and a negative set having the same size.

**Table 4:** Testing results of RhoTermPredict and performances of the Rho-independent terminators tool ARNold in the positive and negative set of sequences of *B. subtilis* 168^a^

Tool	TP	FN	FP	TN	Precision (%)	Recall (%)	Specificity (%)	Accuracy (%)	F_1_-score
RhoTermPredict	17	17	5	29	77.3	50.0	85.3	67.5	0.6
ARNold	4	30	1	33	80.0	11.8	97.0	54.4	0.2

^a^Test experiments were repeated 10 times for 34 randomly selected sequences of negative sets of *B. subtilis* 168 (in order to have a positive and a negative set of the same size), and the means were taken

The F_1_-score obtained by RhoTermPredict was 0.6, lower than that obtained for *E. coli* K-12. Precision, accuracy and specificity were high, while recall was only 50%. Nevertheless we could conclude that the performances of RhoTermPredict with the genomic sequences of *B. subtilis* 168 were good, taking into account that RhoTermPredict is the first reported tool to be able to perform this analysis on a single pipeline. Furthermore, also in genomic regions of *B. subtilis* 168 in which Rho-dependent terminators were expected, ARNold tool was not performing (F_1_-score 0.2, Table 4).

## Discussion

In this study we investigated the possibility to identify Rho-dependent terminator elements through the detection of canonical features previously identified in this class of terminators [5, 9, 28]. We introduced RhoTermPredict, a novel algorithm that predicts putative Rho-dependent transcription terminators based on three indispensable features: i.) 78-nt long RUT site with C > G content and ii.) cytosine spacing (every 11–13 nt); iii.) a possible pausing site for RNAP, precisely hairpin structures, downstream from the putative RUT site. The evaluation of RhoTermPredict performances on *E. coli* K-12 and *B. subtilis* 168 genomes showed that it could be a powerful tool on predicting Rho-dependent terminators. In fact, by using the positive and the negative set of sequences (see Implementation section for details), the F_1_-scores obtained were about 0.7 and 0.6 (Tables 2 and 4), an excellent result taking into account the difficulty of the problem and that RhoTermPredict is the first published algorithm for the prediction of Rho-dependent terminators in a complete way.

In order to have a further demonstration of RhoTermPredict efficacy, we tested it also on the 104 genomic sequences of *E. coli* K-12 substr. MG1655 (U00096.3) (54 sequences) and *S. enterica* LT2 (NC 003197.1) (50 sequences) used by [26] (mean length > 500 nt). These sequences were divided into the 3 classes “None” (32 genomic sequences), “Weak” (38 genomic sequences) and “Strong” (34 genomic sequences) based on the results of the transcription termination experiments, i.e. on the visual changes in the transcription profiles induced by Rho [26]. Class “None” refers to a no action of Rho in the transcription termination experiments; class “Weak” for a weak action of Rho and, finally, class “Strong” for a strong action of Rho. The total number of sequences of the classes “None”, “Weak” and “Strong” in which RhoTermPredict predicted at least one putative Rho-dependent terminator were, respectively, 3/32 (about 9%), 25/38 (about 66%) and 30/34 (about 89%). From this analysis it is clear that RhoTermPredict has a high specificity-value because the FP% rate was only the 9%, while it is more sensitive the stronger is the action of Rho.

Also in the genome-wide analysis we observed good performances for RhoTermPredict. In fact the majority of obtained predictions in the whole genome of *E. coli* K-12 were in regions in which there is a negative gradient of read count value by RNA-Seq data, especially for predictions in IRs, where the percentage of validated predictions was about 70.5 (about 62.4% for all predictions) (Table 3). These good results indicated that RhoTermPredict could also be seen as a genome-wide predictor. It should be noticed that transcriptional gradients are impacted by nutritional conditions that may affect translation/transcription coupling and, as a consequence, premature Rho-dependent transcription termination [10]. However, these effects should not be relevant to our analysis since they would generate, at most, an underestimation of the actual number of Rho-dependent transcription terminators.

**Fig. 5:**
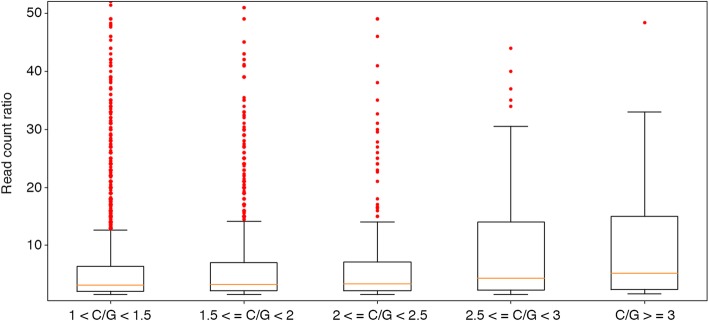
Boxplot of the predictions read value ratios obtained for various window of the C/G ratio values

Our analysis also provided us with the opportunity to analyze, on a genomic scale, a number of features of the putative Rho-dependent terminators. No strong correlation was observed between C/G ratio and RNA-Seq read value ratio but, intriguingly, all the read value ratios > 100 were obtained for C/G ratios < 2 (Table 4 and Additional file 3). This finding could apparently indicate that features such as high C/G ratio and, hence, lowly structured RUT site are not necessarily correlated with termination efficiency, consistently with in vitro results indicating that termination efficiency at a Rho-dependent terminator is an inverse function of the rate of elongation of RNA polymerase [44], and with both in vitro and in vivo results demonstrating that the efficiency depends on kinetic coupling between RNA polymerase and Rho by the “tethered tracking” mechanism [42, 43]. However, a further analysis demonstrated that the previous 46 read value ratios > 100 were a consequence of high expressed upstream genes (Additional file 4). In fact, stronger is a promoter (and, hence, more expressed is a genome region), higher is the read value negative gradient by RNA-Seq data near a terminator. On the contrary, we observed that the terminator strength tends to increase as the C/G ratio increases. In Fig. 5, we showed the boxplots of the predictions read value ratios obtained for various window of the C/G ratio values, where it is possible to notice that the median of distribution tends to slowly increase as the C/G ratio increases (Table 5), despite the highest values were obtained for low C/G ratios (Fig. 5, and Additional file 3: Figure S1). All the statistical informations about validated predictions from RNA-Seq data were reported in Table 4. Therefore we could conclude that as the C/G content of the RUT site increases, Rho binding on RNA transcript is more favorable and, consequently, the termination efficiency increases.

**Table 5:** Statistical informations about validated predictions from RNA-Seq data

	C/G < 1.5	1.5 < = C/G < 2	2 < = C/G < 2.5	2.5 < = C/G < 3	C/G > = 3
Number of predictions	2930	1206	254	67	37
Read value ratios mean	13.3	9.3	7	10.2	10.5
Read value ratios std	168.1	25.7	9.4	13.1	10.5
Read value ratios median	3.2	3.3	3.4	4.4	5.2
Read value ratios > 100	33	13	0	0	0

The number of predictions, the mean, the median and the standard deviation of read value ratios were reported for various window of the predicted RUT site C/G ratio values

Finally, we evaluated the degree of overlap between Rho-dependent and intrinsic terminators by running ARNold tool [25, 29], that predicts intrinsic terminators, both in the same positive and negative sets of sequences (see Implementation section for details), used to test RhoTermPredict performances, and in the whole genome of *E. coli* K-12. Testing results for ARNold tool in positive and negative sets of sequences were not good (F_1_-score obtained was about 0.1) and we expected this because ARNold tool is not suitable for a Rho-dependent terminators search. Furthermore, we compared the predictions obtained by the two programs in the whole genome of *E. coli* K-12 and we observed that only 23% of ARNold predictions overlapped with a RhoTermPredict prediction. These results demonstrated that the degree of overlap between RhoTermPredict and ARNold predictions is very low and, hence, that intrinsic and Rho-dependent terminations are different mechanisms to terminate the DNA transcription.

## Conclusions

Based on our outcomes, we could conclude that the algorithm RhoTermPredict is specific on searching for Rho-dependent terminators and could be used in combination with an intrinsic terminators prediction tool/program for the prediction of all transcription terminators in a bacterial genome. The action of Rho is largely unknown in most microorganisms; in fact the Rho-dependent transcription termination was studied in a depth and exhaustive way only on *E. coli*, *S. enterica* and *B. subtilis*. For this reason an exhaustive list of putative Rho-dependent terminators, necessary for the creation of the positive set for the validation of the algorithm, is available only for the three previous analyzed genomes and, so, we could not use other genomes. But we could say that RhoTermPredict is certainly performant in *E. coli*, *S. enterica* and *B. subtilis*, and possibly in other genomes where Rho-dependent transcription termination occurs. The code of RhoTermPredict is also available within the Additional file 5 of this study.

## Additional files

Additional file 1: Coordinates of the Rho-dependent terminators predicted by RhoTermPredict in *E. coli* K-12 whole genome. (XLSX 621 kb)

Additional file 2: Informations about whole genome predictions by RhoTermPredict from *E. coli* K-12. (TXT 20868 kb)

Additional file 3: **Figure S1.** Correlation analysis between RNAseq read value ratios and C/G content of putative RUT site of validated genome-wide predictions. (DOCX 296 kb)

Additional file 4: List of predictions, with relative genes, characterized by read value ratios > 100. (XLSX 14 kb)

Additional file 5: RhoTermPredict algorithm. (PY 15 kb)

## Abbreviations

BST: Bicyclomycin significant transcript
CDS: Coding sequence
FN: False negatives
FP: False positives
IR: Intergenic region
RNAP: RNA polymerase
RUT: Rho utilization
TN: True negatives
TP: True positives

## References

[1] Ray-Soni A, Bellecourt MJ, Landick R (2016). Mechanisms of bacterial transcription termination: all good things must end. Annu Rev Biochem.

[2] d’Aubenton Carafa Y, Brody E, Thermes C (1990). Prediction of rho-independent *Escherichia coli* transcription terminators. A statistical analysis of their RNA stem-loop structures J Mol Biol.

[3] Ciampi MS (2006). Rho-dependent terminators and transcription termination. Microbiology..

[4] Banerjee S, Chalissery J, Bandey I, Sen R (2006). Rho dependent transcription termination: more questions than answers. J Microbiol.

[5] Mitra P, Ghosh G, Hafeezunnisa M, Sen R (2017). Rho protein: roles and mechanisms. Annu Rev Microbiol.

[6] Richardson JP (2002). Rho-dependent termination and ATPases in transcript termination. Biochim Biophys Acta.

[7] Hart CM, Roberts JW (1994). Deletion analysis of the lambda tR1 termination region: effect of sequences near the transcript release sites and the minimum length of Rho-dependent transcripts. J Mol Biol.

[8] Alifano P, Ciampi MS, Nappo AG, Bruni CB, Carlomagno MS (1988). 1988. In vivo analysis of the mechanisms responsible for strong transcriptional polarity in a "sense" mutant within an intercistronic region. Cell..

[9] Alifano P, Rivellini F, Limauro D, Bruni CB, Carlomagno MS (1991). A consensus motif common to all rho-dependent prokaryotic transcription terminators. Cell..

[10] Alifano P, Rivellini F, Nappo AG, Bruni CB, Carlomagno MS (1994). Alternative patterns of *his* operon transcription and mRNA processing generated by metabolic perturbation. Gene..

[11] Peters JM, Vangeloff AD, Landick R (2011). Bacterial transcription terminators: the RNA 3′-end chronicles. J Mol Biol.

[12] Epshtein V, Dutta D, Wade J, Nudler E (2010). An allosteric mechanism of rho-dependent transcription termination. Nature..

[13] Koslover DJ, Fazal FM, Mooney RA, Landick R, Block SM (2012). Binding and translocation of termination factor rho studied at the single-molecule level. J Mol Biol.

[14] Boudvillain M, Figueroa-Bossi N, Bossi L (2013). Terminator still moving forward: expanding roles for rho factor. Curr Opin Microbiol.

[15] Grylak-Mielnicka A, Bidnenko V, Bardowski J, Bidnenko E (2016). Transcription termination factor rho: a hub linking diverse physiological processes in bacteria. Microbiology..

[16] Kriner MA, Sevostyanova A, Groisman EA (2016). Learning from the leaders: gene regulation by the transcription termination factor rho. Trends Biochem Sci.

[17] Nicolas P, Mader U, Dervyn E, Rochat T, Leduc A, Pigeonneau N (2012). Condition-dependent transcriptome reveals high-level regulatory architecture in *Bacillus subtilis*. Science..

[18] Peters JM, Mooney RA, Grass JA, Jessen ED, Tran F, Robert Landick R (2012). Rho and NusG suppress pervasive antisense transcription in *Escherichia coli*. Genes Dev.

[19] Botella L, Vaubourgeix J, Livny J, Schnappinger D (2017). Depleting *Mycobacterium tuberculosis* of the transcription termination factor rho causes pervasive transcription and rapid death. Nat Commun.

[20] Mader U, Nicolas P, Depke M, PaneÂ-FarreÂ J, Debarbouille M (2016). Van der Kooi-pol M. M, et al. *Staphylococcus aureus* transcriptome architecture: from laboratory to infection-mimicking conditions. PLoS Genet.

[21] Bidnenko V, Nicolas P, Grylak-Mielnicka A, Delumeau O, Auger S, Aucouturier A, Guerin C, Repoila F, Bardowski J, Aymerich S, Bidnenko E (2017). Termination factor rho: from the control of pervasive transcription to cell fate determination in *Bacillus subtilis*. PLoS Genet.

[22] Kingsford CL, Ayanbule K, Salzberg SL (2007). Rapid, accurate, computational discovery of rho-independent transcription terminators illuminates their relationship to DNA uptake. Genome Biol.

[23] Gardner PP, Barquist L, Bateman A, Nawrocki EP, Weinberg Z (2011). RNIE: genome-wide prediction of bacterial intrinsic terminators. Nucleic Acids Res.

[24] Solovyev V, Salamov A, Li RW (2011). Automatic annotation of microbial genomes and metagenomic sequences. Metagenomics and its applications in agriculture, biomedicine and environmental studies.

[25] Naville M, Ghuillot-Gaudeffroy A, Marchais A, Gautheret D (2011). ARNold: a web tool for the prediction of rho-independent transcription terminators. RNA Biol.

[26] Nadiras C, Eveno E, Schwartz A, Figueroa-Bossi N, Boudvillain M (2018). A multivariate prediction model for rho-dependent termination of transcription. Nucl Acids Res.

[27] Di Salvo M, Pinatel E, Talà A, Fondi M, Peano C, Alifano P (2018). G4PromFinder: an algorithm for predicting transcription promoters in GC-rich bacterial genomes based on AT-rich elements and G-quadruplex motifs. BMC Bioinformatics.

[28] Rivellini F, Alifano P, Piscitelli C, Blasi V, Bruni CB, Carlomagno MS (1991). A cytosine- over guanosine-rich sequence in RNA activates rho-dependent transcription termination. Mol Microbiol.

[29] ARNold webtool. http://rna.igmors.u-psud.fr/toolbox/arnold/. Accessed 20 May 2018.

[30] Gautheret D, Lambert A (2001). Direct RNA motif definition and identification from multiple sequence alignments using secondary structure profiles. J Mol Biol.

[31] Lesnik EA, Sampath R, Levene HB, Henderson TJ, McNeil JA, Ecker DJ (2001). Prediction of rho-independent transcriptional terminators in *Escherichia coli*. Nucleic Acids Res.

[32] Python. https://www.python.org. Accessed 22 Apr 2018.

[33] Blattner FR, Plunkett G, Bloch CA, Perna NT, Burland V, Riley M, Collado-Vides J, Glasner JD, Rode CK, Mayhew GF, Gregor J, Davis NW, Kirkpatrick HA, Goeden MA, Rose DJ, Mau B, Shao Y (1997). The complete genome sequence of *Escherichia coli* K-12. Science..

[34] Bear DG, Hicks PS, Escudero KW, Andrews CL, McSwiggen JA, von Hippel PH (1988). Interactions of Escherichia coli transcription termination factor rho with RNA. II Electron microscopy and nuclease protection experiments. J Mol Biol.

[35] Modrak D, Richardson JP (1994). The RNA-binding domain of transcription termination factor rho: isolation, characterization, and determination of sequence limits. Biochemistry..

[36] Morgan WD, Bear DG, Litchman BL, von Hippel PH (1985). RNA sequence and secondary structure requirements for rho-dependent transcription termination. Nucl Acids Res..

[37] Richardson JP (2003). Loading rho to terminate transcription. Cell..

[38] Dar D, Sorek R (2018). High-resolution RNA 3′-ends mapping of bacterial rho-dependent transcripts. Nucleic Acids Res.

[39] Herbert KM, La Porta A, Wong BJ, Mooney RA, Neuman KC, Landick R, Block SM (2006). Sequence-resolved detection of pausing by single RNA polymerase molecules. Cell..

[40] Larson MH, Mooney RA, Peters JM, Windgassen T, Nayak D, Gross CA, Block SM, Greenleaf WJ, Landick R, Weissman JS (2014). A pause sequence enriched at translation start sites drives transcription dynamics in vivo. Science..

[41] Vvedenskaya IO, Vahedian-Movahed H, Bird JG, Knoblauch JG, Goldman SR, Zhang Y, Ebright RH, Nickels BE (2014). Interactions between RNA polymerase and the “core recognition element” counteract pausing. Science..

[42] Gocheva V, Le Gall A, Boudvillain M, Margeat E, Nollmann M (2015). Direct observation of the translocation mechanism of transcription termination factor rho. Nucleic Acids Res.

[43] Jin DJ, Burgess RR, Richardson JP, Gross CA (1992). Termination efficiency at rho-dependent terminators depends on kinetic coupling between RNA polymerase and rho. Proc Natl Acad Sci U S A.

[44] Richardson LV, Richardson JP (1996). Rho-dependent termination of transcription is governed primarily by the upstream rho utilization (rut) sequences of a terminator. J Biol Chem.

