# Supplementary material for: RhoTermPredict: an algorithm for predicting Rho-dependent transcription terminators based on Escherichia coli, Bacillus subtilis and Salmonella enterica databases
Source: BMC Bioinformatics. 2019 Mar 7;20:117. doi: 10.1186/s12859-019-2704-x (PMC6407284; doi:10.1186/s12859-019-2704-x)
Supplement: Supplementary file 3 — Figure S1. Correlation analysis between RNAseq read value ratios and C/G content of putative RUT site of validated genome-wide predictions. (DOCX 296 kb) [file 12859_2019_2704_MOESM3_ESM.docx]

**
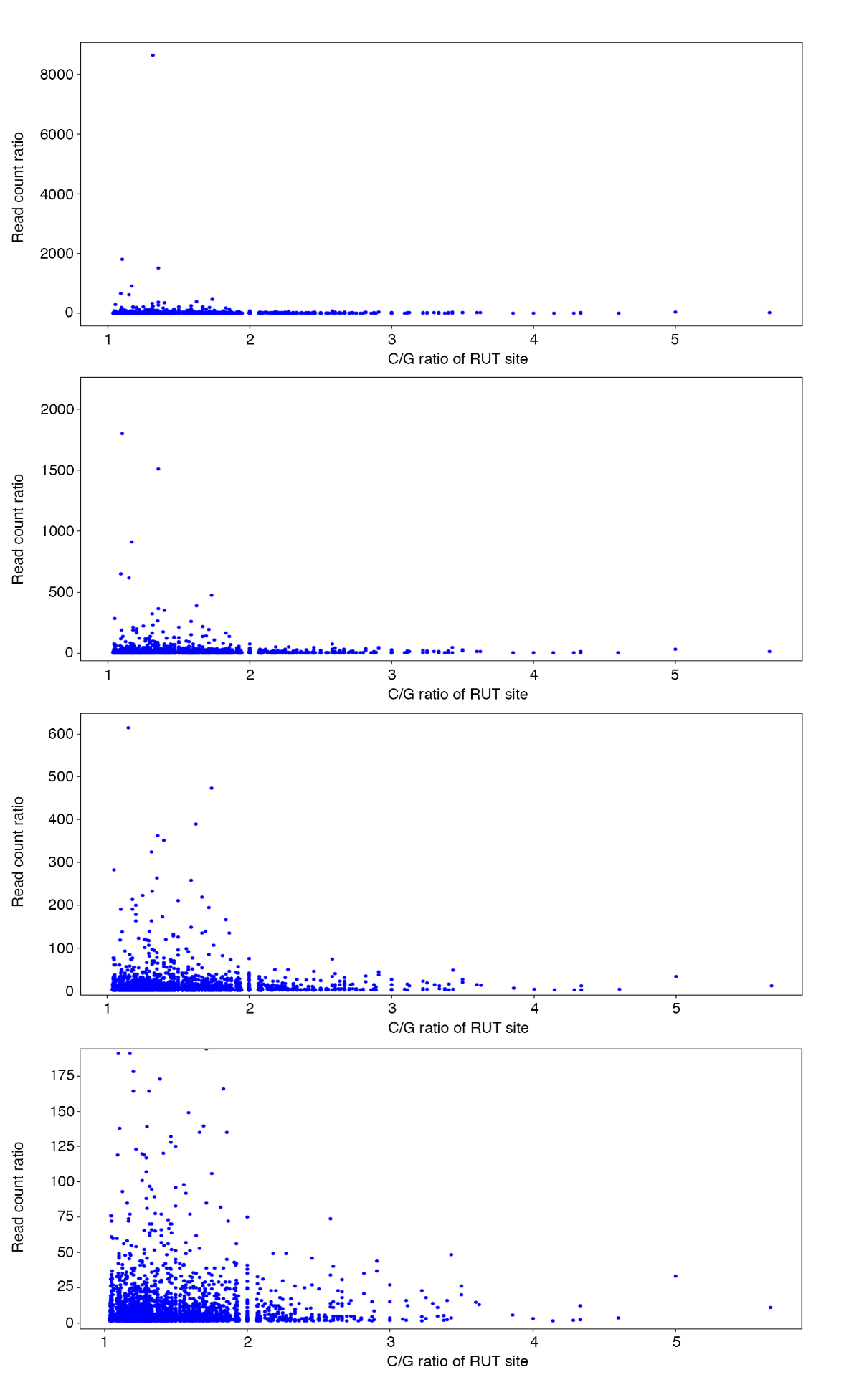
**

**Figure S1** Correlation analysis between RNA-Seq read count ratios and C/G content of putative RUT site of validated genome-wide predictions, for different scales of read count ratio values.
